# Liquid Oxygen Compatibility and Ultra-Low-Temperature Mechanical Properties of Modified epoxy Resin Containing Phosphorus and Nitrogen

**DOI:** 10.3390/polym14204343

**Published:** 2022-10-15

**Authors:** Ni Liu, Hui Wang, Shun Wang, Baosheng Xu, Lijie Qu

**Affiliations:** 1 Institute of Advanced Structure Technology, Beijing Institute of Technology, Beijing 100081, China; 2 Beijing Key Laboratory of Lightweight Multi-Functional Composite Materials and Structures, Beijing Institute of Technology, Beijing 100081, China

**Keywords:** epoxy resin, BSEA, flame retardant, liquid oxygen compatibility, mechanical properties

## Abstract

Endowing epoxy resin (EP) with prospective liquid oxygen compatibility (LOC) as well as enhanced ultra-low-temperature mechanical properties is urgently required in order to broaden its applications in aerospace engineering. In this study, a reactive phosphorus/nitrogen-containing aromatic ethylenediamine (BSEA) was introduced as a reactive component to enhance the LOC and ultra-low-temperature mechanical properties of an EP/biscitraconimide resin (BCI) system. The resultant EP thermosets showed no sensitivity reactions in the 98J liquid oxygen impact test (LOT) when the BSEA content reached 4 wt% or 5 wt%, indicating that they were compatible with liquid oxygen. Moreover, the bending properties, fracture toughness and impact strength of BSEA-modified EP were greatly enhanced at RT and cryogenic temperatures (77 K) at an appropriate level of BSEA content. The bending strength (251.64 MPa) increased by 113.67%, the fracture toughness (2.97 MPa·m^1/2^) increased by 81.10%, and the impact strength (31.85 kJ·m^−2^) increased by 128.81% compared with that of pure EP at 77 K. All the above results demonstrate that the BSEA exhibits broad application potential in liquid oxygen tanks and in the cryogenic field.

## 1. Introduction

The requirements for lightweight structures in large launch vehicles are increasing with the development of future major space missions such as deep space exploration and interstellar travel [1,2,3]. Liquid oxygen propellant tanks are the main weight reduction components as the tanks are of considerable weight and volume within large launch vehicles [4,5]. With the evolution of carbon-fiber-reinforced resin matrix composites (CFRP) and their large-scale application in aerospace engineering, the preparation of linerless composite liquid oxygen cryotanks has become an important trend in lightweight spacecraft [6,7,8,9,10]. The tank weights could be reduced by 20–40% if using CFRP instead of a conventional alloy material, because CFRP have low density, a superior specific modulus and strength and outstanding resistance to fatigue, which considerably improves the flight efficiency of the space vehicle [11,12,13,14,15]. EP is a common high-performance resin matrix utilized in the field of cryogenic engineering, including the development of liquid oxygen tanks, because of its desirable properties, such as chemical resistance, good dimensional stability, excellent processability and mechanical properties [16,17,18,19,20,21]. However, risks exist, such as the fact that EP is likely to explode and spark if exposed to frictional heating, mechanical shock, collision, static electricity, etc., when present in a liquid oxygen environment [2,22,23]. In addition, the brittleness of EP will greatly increase, and impact resistance is reduced substantially in the cryogenic environment [6,24,25]. Therefore, endowing EP thermosets with prospective LOC and improved mechanical properties is urgently required in order to broaden its applications [26].

In particular, 98J LOT is the most commonly utilized method to study the LOC of EP [27]. There are two main theories, namely the hot spot and free radical theories, to explain the incompatibility between polymers and liquid oxygen. Bowden et al. [28] proposed that the mechanical energy could be converted to thermal energy, which is focused on a certain area (called a hot spot) under the shock of liquid oxygen and causes an excessive temperature rise in the area. Amwster et al. [29] believed that active free radicals could be produced on the surface of the polymer; then, the chain reaction between these active free radicals and liquid oxygen could release heat and thus cause charring, flash, explosion and even burning. Fundamentally, the nature of EP’s incompatibility with liquid oxygen is consistent with its violent oxidation reaction. Therefore, enhancing the flame retardancy as well as thermal stability of EP would be a highly efficient approach to improve the LOC.

Phosphorus-containing (P-containing) fire retardants have been widely used to modify EP due to its outstanding flame retardancy, low toxicity and environmentally friendly and non-halogen nature. In recent years, 9,10-dihydro-9-oxa-10-phosphaphenanthrene-10-oxide (DOPO) has been the most prominent P-containing fire retardant for EP because of its superior reaction activity and strong molecular designability [30,31,32,33]. Thus, a previous study proved that not only DOPO, but DOPO derivatives, could improve the LOC of EP as well. Wu et al. [34] utilized DOPO to graft the chemical structures of bisphenol A type EP (EP-A) and bisphenol F type EP, and the LOC and flame retardancy were significantly enhanced, but the mechanical properties of the system have not been studied in depth. Li et al. [35] synthesized EP modified by 10-(2,5-dihydroxyphenyl)-9,10-dihydro-9-oxa-10-phosphaphenanthrene-10-oxide (ODOPB) and EP-A, and the enhanced flame retardancy and thermal stability were proven to improve the LOC of EP, but the mechanism of ODOPB affecting the LOC has not been analyzed in depth. Wang et al. [36] introduced a hyperbranched compound containing phosphorus, silicon elements and boehmite into EP; the results showed that the modified EP could achieve LOC, but the tensile properties of the modified EP at 90 K were decreased and the fracture toughness has not been studied. Li et al. [27] utilized DOPO-PGE to modify EP, and the results indicated that DOPO-PGE was a promising additive in the field of enhancing the LOC of the EP matrix and CFRP, but the mechanical properties of DOPO-PGE-modified EP need further research. Previous studies have investigated various methods to enhance LOC; however, the mechanism of flame retardants affecting the LOC of EP needs further study and the comprehensive mechanical properties need to be further enhanced.

In the authors’ previous research, a reactive phosphorus-/nitrogen-containing fire retardant (BSEA) was synthesized by a facile addition reaction between *N*, *N*’-bis(salicylidene)ethylenediamine (NEA) and DOPO with high productivity [37]. BSEA was utilized as a multifunctional component to endow an EP/biscitraconimide resin (BCI) system with the expected flame retardancy and desirable tensile properties at RT and 77 K. This inspired us to apply this simple and effective flame retardant to the preparation of liquid oxygen tank materials, which is a very attractive research area. In the authors’ current study, BSEA was introduced to enhance the LOC of EP and enhance the cryogenic mechanical properties. The LOC of EP composites was detected by 98J LOT, and the essence of BSEA affecting the LOC was analyzed in depth, combined with the authors’ previous work. Moreover, the mechanical properties, such as bending properties and fracture toughness (K_IC_), as well as the impact property, of the EP composites in the cryogenic environment were analyzed in detail and contrasted with those at RT.

## 2. Materials and Methods

### 2.1. Materials

EP-A (E44, epoxy value 0.51–0.54) was selected as the EP matrix provided by Baling Petrochemical Co., Ltd. (Yueyang, China). DOPO was purchased from Meryer Chemical Technology Co., Ltd. (Shanghai, China). BCI was supplied by Jiataixing Plastic Technology Co., Ltd. (Dongguan, China). BSEA was obtained from our previously synthesized flame-retardant [37]. 4,4′-diaminodiphenylmethane (DDM) was available from Aladdin Biochemical Technology Co., Ltd. (Shanghai, China).

### 2.2. Experimental Methods—Preparation of BSEA-Modified Epoxy Resin

**Figure 1:**
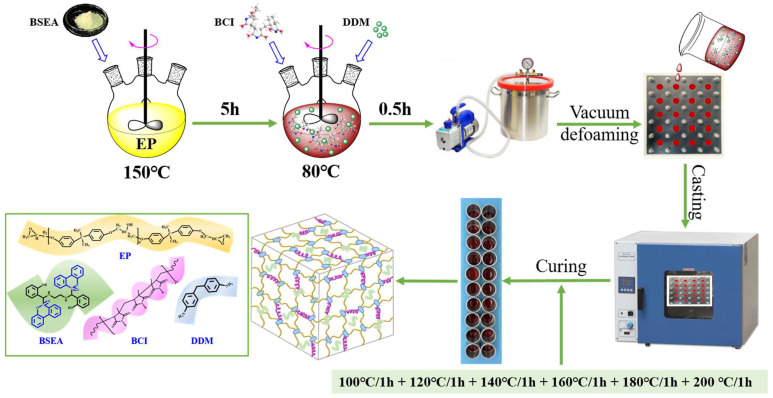
Schematic of the preparation process and crosslinking network of the EP composites.

The preparation steps of EP composites containing different mass ratios (2, 3, 4 and 5 wt%) of BSEA followed by the routes presented in Figure 1, and the formulations of these composites are shown in Table 1. First, the calculated amount of BSEA and EP was mixed under vigorous stirring at 150 °C for 5 h to ensure adequate dispersion and reaction. Then, the mixture was lowered to 80 °C and BCI was introduced to the above blend to continue stirring until a transparent and homogeneous blend was obtained. Next, the calculated DDM was introduced to the resulting blend and stirred at 80 °C for 30 min to obtain the prepolymer. After vacuum defoaming, the prepolymer was poured into a rustless steel mold which was preheated and sprayed with 820-NC releasing agent for curing. Lastly, the stainless-steel mold was put into the oven for curing and the gradient curing process of 100 °C/1 h + 120 °C/1 h + 140 °C/1 h + 160 °C/1 h + 180 °C/1 h + 200 °C/1 h was carried out to obtain the cured EP composites. The crosslinking network schematic of modified EP is presented in Figure 1. Moreover, the preparation of pure EP and EP/BCI-25 specimens was the same as in our previous work [1].

### 2.3. Characterization Methods

The LOC was tested on an Army Ballistic Missile Agency (ABMA)-type impact tester under 98 J impact energy, referring to ASTM D2512-9. X-ray photoelectron spectroscopy (XPS) was performed on Thermo Scientific K-Alpha (Thermo Fisher Scientific, Waltham, MA, USA) to detect C, O, N and P elements before and after liquid oxygen impact. Scanning electronic microscopy (SEM) was performed on JSM-7001F equipment (JEOL, Tokyo, Japan) to study the fracture morphology and char residues. The surfaces of the samples were cleaned with ethanol before testing and a layer of carbon was sprayed to improve electrical conductivity. The automatic vacuum carbon injection instrument was utilized to spread a carbon layer onto the surface of specimens. The carbon injection process mainly consists of installing carbon rod, putting it into the sample table with the sample to be tested, vacuuming and carbon injection.

The bending properties were tested with the INSTRON 5967 electronic universal material testing machine (INSTRON, Norwood, MA, USA) at a loading rate of 2 mm/min. The bending samples were shaped into strips with the dimensions of 60 mm × 15 mm × 3 mm, referring to GB/T 2567-2008. Single-edge notch bend (SENB) test was measured on same equipment to determine fracture toughness (*K_IC_*) at a speed of 1 mm/min referred to ASTM D5045-99. SENB samples were shaped into cuboids with dimensions of 44 mm × 10 mm × 5 mm, and the notch and crack were prefabricated before the test. The *K_IC_* value was calculated according to the following formula:

(1) $$K_{IC}=\frac{SP_{Q}}{DW^{3/2}}f\left(\frac{a}{W}\right)$$

in which *f*(*a/W*) is calculated referring to the following formular:

(2) $$f\left(\frac{a}{W}\right)=\frac{3{\left(a/W\right)}^{1/2}\left[1.99-\left(a/W\right)\left(1-a/W\right)\times \left(2.15-3.93a/W+2.7a^{2}/W^{2}\right)\right]}{2\left(1+2a/W\right){\left(1-a/W\right)}^{3/2}}$$

in which *P_Q_* represents the break load, *S* represents the span (40 mm), *f* represents the geometrical correction coefficient of the polynomial, *D* and *W* represent the sample thickness (5 mm) and sample width (10 mm), and *a* represents the length (5 mm) of the crack. Impact strength was tested on the RXJ-50 impact tester (China) with sample dimensions of 80 mm × 10 mm × 4 mm, referring to GB/T 2567-2008. At least five samples of each component were prepared for parallel testing.

## 3. Results and Discussion

### 3.1. Liquid Oxygen Compatibility of EP/BCI/BSEA Composites

#### 3.1.1. Results of Liquid Oxygen Compatibility

**Figure 2:**
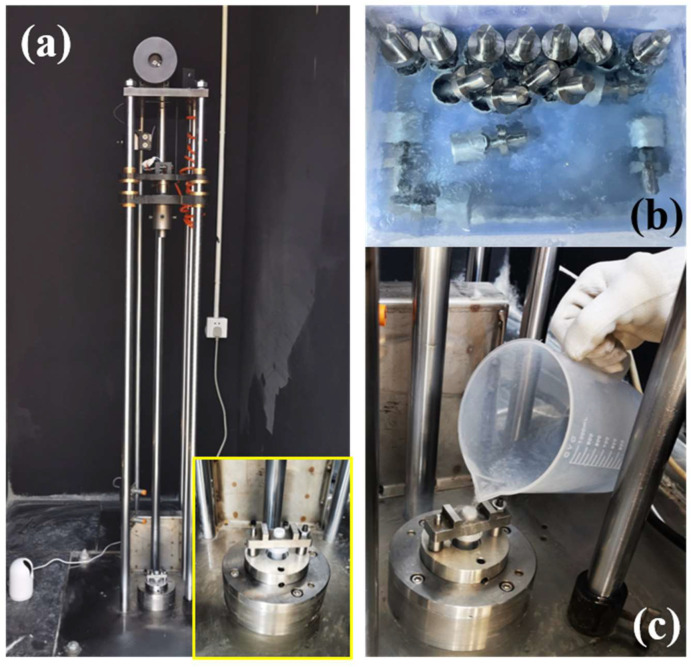
(**a**) 98J LOT machine with high-definition camera, (**b**) samples, specimen cup and striker pin immersed in liquid oxygen and (**c**) liquid oxygen poured into the specimen cup before impact.

**Figure 3:**
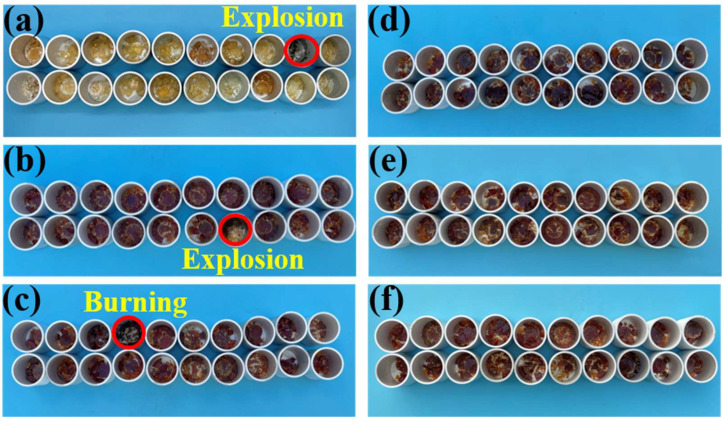
Schematic of (**a**) pure EP, (**b**) EP/BCI-25, (**c**) EP/BCI/BSEA-2, (**d**) EP/BCI/BSEA-3, (**e**) EP/BCI/BSEA-4, (**f**) EP/BCI/BSEA-5 after impact in liquid oxygen.

The LOC of EP thermosets was evaluated by 98J LOT, and the corresponding testing steps were as follows. Firstly, the cured EP specimen was placed into the sample cup and pre-cooled in a container of liquid oxygen along with the cup and impact column. Secondly, the sample cup was placed into the cup holder focused on the anvil section assembly of the machine, and we next placed the pre-cooled impact pin in the center of the cup. Lastly, the hammer plummeted from a height of 1.1 m at free fall onto the column, which transferred the mechanical energy to the samples. Views of all phenomena (including burning, explosion, charring, flash) were recorded during the test, and the LOT sensitivity of the samples was recorded. The sample was considered to be compatible with the liquid oxygen if none of the above phenomena were observed in 20 tests. Moreover, the impact reaction sensitivity coefficient (IRS) was utilized to analyze the LOC of EP. The calculation of IRS was performed according to the following formula [2,27,38].

(3) $$IRS=\frac{\sum_{i}^{4}w_{i}n_{i}}{N}$$

in which *N* represents the total test time, *n_i_* represents the frequency of the reaction, *w_i_* represents the weight coefficient of different phenomena, namely burning (*w*_1_ = 1), explosion (*w*_2_ = 0.9), flash (*w*_3_ = 0.6), charring (*w*_4_ = 0.4). Figure 2 shows the test equipment and the preparation before the impact test, and Figure 3 shows digital photos of all samples after impact in liquid oxygen. As summarized in Table 2, the IRS of pure EP and EP/BCI-25 were both 10.5%, indicating incompatibility with liquid oxygen and high reaction intensity between the two cured EP samples and liquid oxygen. The IRS of the EP/BCI/BSEA-2, with one burning and one charring instance, was 7.0%, and the EP/BCI/BSEA-3 with two charring instances was 4.0%, which indicated that the IRS decreased with the addition of BSEA. The videos of the burning (Video S1) and the lack of reactions of EP/BCI/BSEA-2 during the impact tests (Video S2) are included in the Supporting Information. Furthermore, the EP thermosets exhibited no sensitivity phenomena in 20 impact tests when the BSEA content reached 4 wt% or 5 wt%, indicating that the two cured EP samples achieved LOC. The results are consistent with the flame-retardant performance of BSEA-modified EP [37], revealing that improving the flame retardancy property is a highly effective approach to enhance the LOC of EP.

#### 3.1.2. XPS Analysis before and after 98J LOT

**Figure 4:**
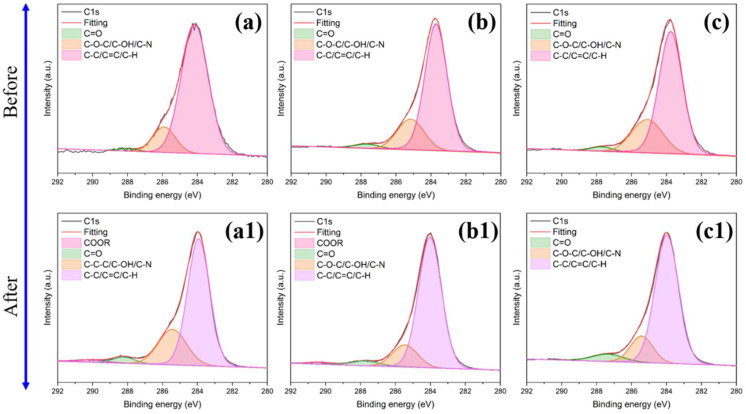
C1s spectra of pure EP (**a**) before and (**a1**) after, EP/BCI/BSEA-2 (**b**) before and (**b1**) after, EP/BCI/BSEA-5 (**c**) before and (**c1**) after the liquid oxygen compatibility test.

XPS was conducted to research the elemental composition of EP composites before and after 98J LOT and explore the mechanism of BSEA affecting the LOC of EP. The C1s spectra of pure EP, EP/BCI/BSEA-2 and EP/BCI/BSEA-5 before and after 98J LOT are exhibited in Figure 4, and the corresponding peak fitting values are summarized in Table 3. As exhibited in Figure 4(a–c,a1–c1), the C1s peak-differentiating and imitating results before and after 98J LOT at around 283.98 eV, 285.43 eV, 287.99 eV and 289.99 eV were ascribed to C–C/C=C/C–H bonds, C–O–C/C–OH/C–N bonds, a C=O bond and COOR [39,40], respectively. As summarized in Table 3, the C–C/C=C/C–H content of pure EP was decreased after impact, but the C–O–C/C–OH/C–N content and C=O bond content were improved and the COOR appeared. This is probably because C–C/C–H bonds can decompose into carbon radicals and ·H under high-energy mechanical impact, which is more conductive to the combination of oxygen radicals to form C–O–C, C=O and COOR bonds under a liquid oxygen environment. Moreover, the C–O–C/C–OH/C–N content of EP/BCI/BSEA-2 and EP/BCI/BSEA-5 was decreased after impact, but the C–C/C=C/C–H content and C=O content increased. This mainly because the C–OH bond of BSEA-modified EP broke into carbon radicals and ·OH and regenerated C=C and C=O bonds under the oxygen-rich environment.

**Figure 5:**
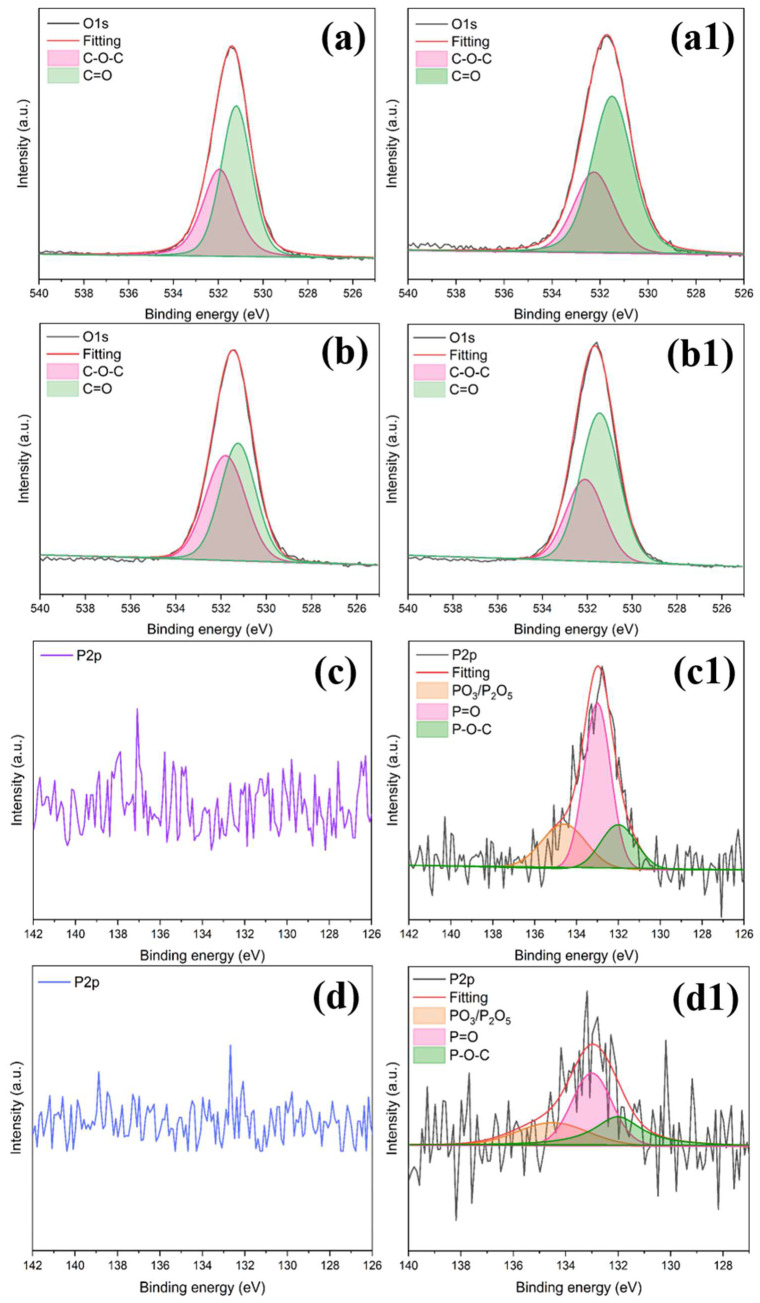
O1s spectra of EP/BCI/BSEA-2 (**a**) before and (**a1**) after, EP/BCI/BSEA-5 (**b**) before and (**b1**) after the liquid oxygen compatibility test, P2p spectra of EP/BCI/BSEA-2 (**c**) before and (**c1**) after, EP/BCI/BSEA-5 (**d**) before and (**d1**) after the liquid oxygen compatibility test.

**Figure 6:**
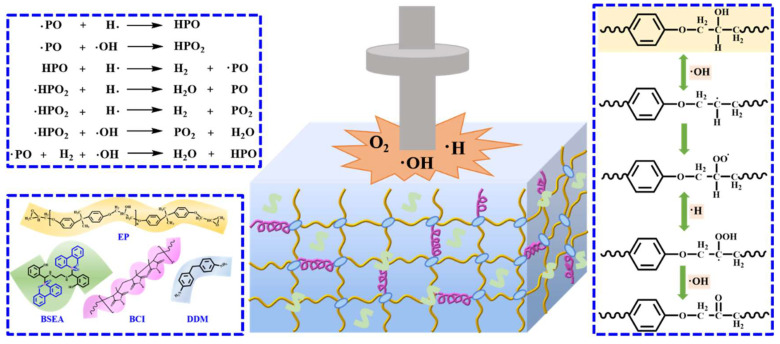
Liquid oxygen compatibility mechanism of the BSEA-modified EP.

The O1s spectra of EP/BCI/BSEA-2 and EP/BCI/BSEA-5 before and after impact are shown in Figure 5, and that of pure EP is shown in Figure S1. The relevant peak fitting values of O1s are shown in Table 4. As exhibited in Figure 5(a,b,a1,b1), the O1s peak-differentiating and imitating results of EP samples before and after liquid oxygen impact at around 531.45 eV and 532.10 eV were attributed to the C=O band and C–O–C [41]. The peak fitting data in Table 4 show that the relative amount of C=O bonds improved, while the amount of C–O–C bonds was reduced. This is mainly attributed to the fact that the R–OH in the molecular structure was attacked under 98J LOT in the cryogenic environment to generate C=O. The specific reaction route is exhibited in Figure 6 on the right. Firstly, the R–OH in the EP matrix was attacked to generate R· and ·OH radicals. Secondly, the R· radicals may react with liquid oxygen to generate ROO· radicals, and the ROO· radicals could release ·H at the same time and combine with ·H to form ·COOH. Moreover, ·COOH radicals could release ·OH radicals to form C=O. Therefore, the generation of C=O bonds might be the key factor affecting the LOC of EP.

The N1s fitting peaks before and after impact at around 398.88 eV, 400.08 eV and 405.78 eV were assigned to C–N, O=C–N and NO_3_, as exhibited in Figure S1(b–d,b1–d1). The fitting peak of NO_3_ only appeared after the impact of pure EP, while it did not appear after the impact of BSEA-modified EP, which indicated that the addition of BSEA could increase the antioxidant activity of EP, and thus improve the LOC of EP.

As exhibited in Figure 5c,d, the P2p spectra of EP/BCI/BSEA-2 and EP/BCI/BSEA-5 could hardly be observed before impact. This is because of the low atomic mass fraction of the phosphorus element in the modified EP. Figure 5c1,d1 show that the P2p fitting peaks after impact at around 132.01 eV, 133.00 eV and 134.5 eV were ascribed to PO_3_/P_2_O_5_, P=O and P–O–C, and the corresponding data are summarized in Table 4 [2]. The results indicate that large numbers of PO· and HPO_2_· active free radicals were generated during the impact, and these phosphorus-containing radicals could capture the ·OH, ·H and ·O high-energy free radicals, which could interrupt the chain decomposition of EP and inhibit incompatible reactions between EP and liquid oxygen, as shown in Figure 6 [42,43,44].

#### 3.1.3. Section Morphology Analysis after the 98J LOT

**Figure 7:**
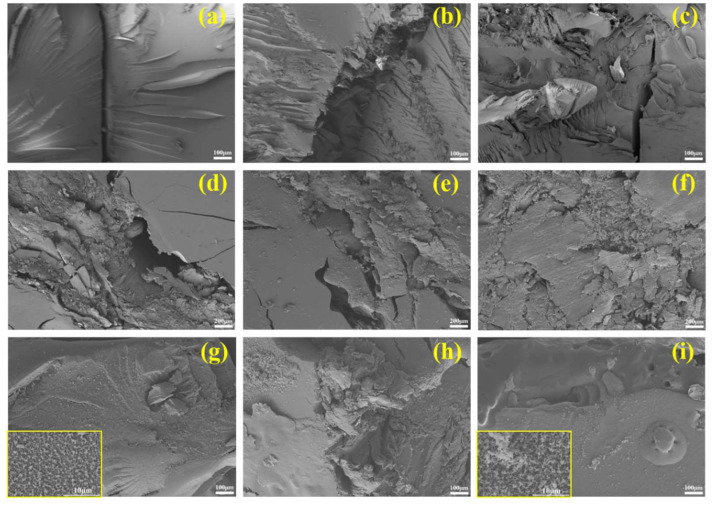
SEM images of section morphology of (**a**) pure EP, (**b**) EP/BCI/BSEA-2 and (**c**) EP/BCI/BSEA-5; surface morphology of (**d**) pure EP, (**e**) EP/BCI/BSEA-2 and (**f**) EP/BCI/BSEA-5 with no reaction; morphology of (**g**) explosion samples, (**h**) flash sample of pure EP and (**i**) burning sample of EP/BCI/BSEA-2 after liquid oxygen compatibility impact test.

The SEM section morphology of samples and surface morphology with no reaction and incompatible reactions after liquid oxygen compatibility tests are presented in Figure 7. As exhibited in Figure 7a–c, the section morphology of EP/BCI/BSEA-2 and EP/BCI/BSEA-5 was obviously rougher than the section morphology of pure EP, indicating that the comprehensive mechanical properties of BSEA-modified EP composites were enhanced, and introducing BSEA could improve the mechanical properties of the EP matrix, which was helpful for inhibiting the occurrence of a sensitive reaction in 98J LOT. As exhibited in Figure 7d–f, the surface topography of pure EP and EP/BCI/BSEA-2 exhibited a fault zone with some crack propagation, but the fault zone in EP/BCI/BSEA-2 was narrower than that of pure EP. Moreover, the surface morphology of the EP/BCI/BSEA-5 sample presented an irregular collapse. The results indicated that EP/BCI/BSEA-2 had excellent impact resistance compared with pure EP and EP/BCI/BSEA-5 samples. Figure 7g shows that there were large amounts of ultrafine particles on the surface of the pure EP sample with the explosion reaction. These ultrafine particles were formed by the deposition of gaseous volatiles released or the incomplete combustion of carbon during the explosion. Figure 7h shows the flash sample of pure EP, which locally presented a similar morphology to the explosion sample. The burning sample [Figure 7i] of EP/BCI/BSEA-2 after the liquid oxygen compatibility impact test showed not only ultrafine particles, but some micropores on the external face of the EP/BCI/BSEA-2 sample. This is mostly due to the mechanism wherein BSEA could generate P-containing high-energy free radicals (PO·, PO_2_· or HPO_2_· radicals) and could be decomposed to a non-flammable gas, such as CO_2_, N_2_, NO_2_ or NH_3_, during the process of burning [37]. These volatile gases break through the carbon layer and exert a flame-retardant effect to form pores, thus preventing further combustion of the EP matrix.

### 3.2. Mechanical Properties of EP/BCI/BSEA Composites

#### 3.2.1. Bending Properties

**Figure 8:**
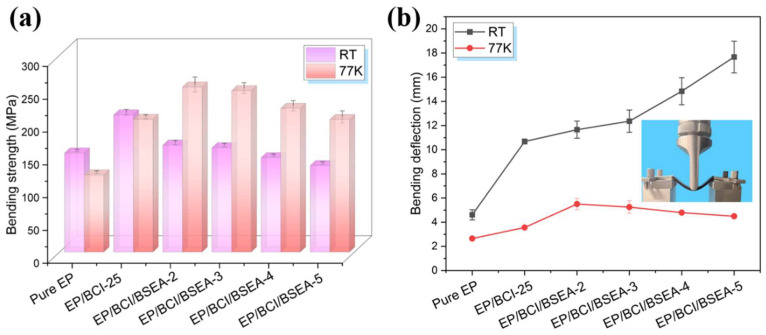
(**a**) Bending strength and (**b**) bending deflection of the EP composites at RT and 77 K.

The bending properties of EP/BCI/BSEA composites are exhibited in Figure 8, and the relevant data are presented in Table S1. As exhibited in Figure 8a and Table S1, the bending strength of EP/BCI/BSEA-2 achieved peak values of 163.10 MPa at RT and 251.64 MPa at 77 K, being 8.04% and 113.67% higher than the peak values of 150.96 MPa at RT and 117.77 MPa at 77 K of pure EP, respectively. The bending strength of BSEA-modified EP was decreased compared with the bending strength of EP/BCI-25, while the bending strengths of EP/BCI/BSEA-2 and EP/BCI/BSEA-3 were still higher than the bending strength of pure EP at RT. This phenomenon was mainly attributed to two main reasons. On one hand, the free volume of BSEA-modified EP crosslinking networks increased with the addition of BSEA content, which led to a decline in crosslinking density [37], thus resulting in the reduction in the bending strength of modified EP. On the other hand, the concentration of reactive functional groups (–OH, –NH) increased with the addition of BSEA in the modified EP system, resulting in an increase in volume shrinkage after curing, which led to increased internal stress in the EP system, and ultimately led to the decrease in bending strength. At an ultra-low temperature, the free volume of EP/BCI/PBAH composites at 77 K was smaller due to thermal shrinking, which led to increased intermolecular force and enhanced bending strength compared with that at RT. The bending strength of the modified EP decreased with the further addition of BSEA, which is similar to the scenario at RT and will not be discussed in detail.

As shown in Table S1, the bending modulus in the cryogenic environment was improved compared with that at RT, which was attributed to the fact that the atomic binding force and intermolecular force in cured EP chains under cryogenic conditions were stronger than those at RT because of thermal shrinking. As summarized in Figure 8a and Table S1, the bending deflection reached the maximum value of 17.67 mm (EP/BCI/BSEA-5) at RT and 5.50 mm (EP/BCI/BSEA-2) at 77 K, achieving an enhancement of 283.30% and 107.55% compared with that of pure EP (4.61 mm at RT and 2.65 mm at 77 K, respectively). The free volume of BSEA-modified EP crosslinking networks increased with the increase in BSEA content, which provided space for segments’ movement, thus resulting in enhanced bending deflection. Moreover, the deflection in bending at 77 K was decreased compared with that at RT for all specimens. This was mostly due to the fact that the free volume at 77 K became smaller than at RT, resulting in the poor mobility of chain segments, and macromolecules were partially frozen at 77 K.

#### 3.2.2. Fracture Toughness

**Figure 9:**
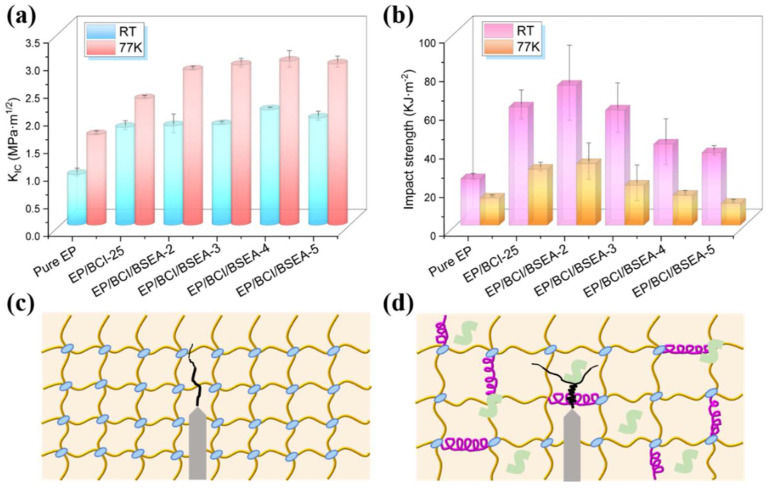
(**a**) *K_IC_* and (**b**) impact strength of EP composites at RT and 77 K; schematic diagram of crack propagation for fracture toughness tests of (**c**) pure EP and (**d**) modified EP.

As exhibited in Figure 9a and Table S2, the *K_IC_* of EP/BCI/BSEA-4 reached a peak value of 2.09 MPa·m^1/2^ at RT and 2.97 MPa·m^1/2^ at 77 K, which showed increases of 127.17% and 81.10% compared with pure EP (0.92 MPa·m^1/2^ at RT and 1.64 MPa·m^1/2^ at 77 K), respectively. This was mainly ascribed to the fact that the plastic deformation capability of modified EP was improved and the microcrack surface due to internal stress was blunted with the addition of BSEA. Moreover, as exhibited in Figure 9c,d, the introduction of BSEA could lead to deflection and disproportionation of the microcrack propagation path compared with pure EP, thus dissipating more fracture energy and enhancing *K*_IC_. In addition, the *K*_IC_ values at 77 K were all higher than those at RT in the same components. This was due to the molecular spacing becoming smaller and the intermolecular force becoming stronger due to thermal contraction under ultra-low temperatures. Furthermore, in addition to intermolecular forces, more major chemical bonds in the crosslinking network needed to be overcome during crack propagation with the introduction of BSEA, which might have resulted in the enhanced *K_IC_* values.

**Figure 10:**
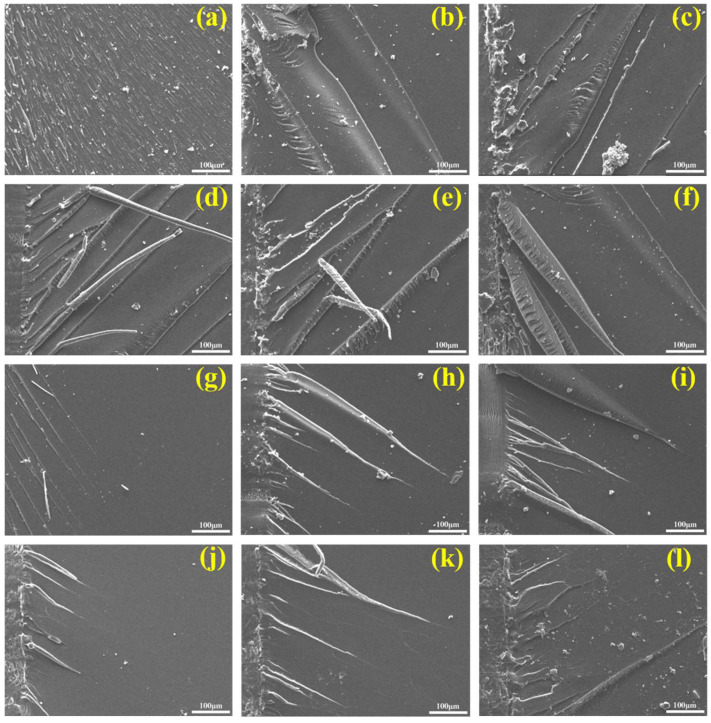
SEM images of the fracture surfaces of the cured EP: (**a**) pure EP, (**b**) EP/BCI-25, (**c**) EP/BCI/BSEA-2, (**d**) EP/BCI/BSEA-3, (**e**) EP/BCI/BSEA-4, (**f**) EP/BCI/BSEA-5 at RT, (**g**) pure EP, (**h**) EP/BCI-25, (**i**) EP/BCI/BSEA-2, (**j**) EP/BCI/BSEA-3, (**k**) EP/BCI/BSEA-4, (**l**) EP/BCI/BSEA-5 at 77 K.

SEM was performed to observe the fracture morphology near the cured EP pre-crack, as described in Figure 10. As presented in Figure 10a–f, the cracking surface topography of pure EP was very smooth at RT, which indicated that pure EP had weak resistance to crack propagation. The cracks became more continuous and rougher than those of pure EP, which was accompanied by stress dissipation streaks, with the introduction of BSEA. In fact, very rough, rodlike cracks appeared in the fracture surface with the further addition of BSEA, as shown in Figure 10d–f. This phenomenon indicated that adding BSEA could improve the crack resistance of EP. As shown in Figure 10g–l, the fracture morphology of pure EP, with obvious linear stripes, was highly smooth at 77 K. The overall appearance of pure EP exhibited obvious brittle fracture characteristics, revealing that the resistance to crack propagation was extremely weak under cryogenic conditions. With the increase in BSEA, the crack section became coarser, indicating that the capability to resist crack propagation was improved at 77 K. Moreover, the cracks at 77 K were shorter than those at RT, indicating that the free volume of the EP/BCI/BSEA crosslinking network under cryogenic conditions was decreased compared with that under RT and the intermolecular bonding force was stronger; thus, the *K_IC_* values at 77 K were enhanced compared with those at RT.

#### 3.2.3. Impact Properties

As summarized in Figure 9b and Table S2, the impact strength of EP/BCI/BSEA-2 achieved a peak value of 72.44 kJ·m^−2^ at RT and 31.85 kJ·m^−2^ at 77 K, with an improvement of 201.83% and 128.81% compared with those of pure EP (24.00 kJ·m^−2^ at RT and 13.92 kJ·m^−2^ at 77 K), respectively. Moreover, the impact strength decreased when the amount of BSEA added continued to increase. The increase in impact strength helped the sample resist local impact forces in the ultra-low-temperature liquid oxygen environment, thereby reducing the impact sensitivity of liquid oxygen and improving the LOC of EP. This result is in line with the conclusion of the previous IRS test. This was mainly due to the mechanism whereby the free volume increased with the addition of BSEA. The free volume could absorb energy by distorting itself, as well as providing a space for molecular chains to move in the crosslinking networks during the process of being impacted by the external forces, and thus increasing the impact strength. However, the microstructural integrity of modified EP could not be maintained because too much BSEA was introduced into the crosslinking networks, leading to a decline in impact strength. Moreover, the impact strengths at 77 K were all decreased compared with those at RT in the same components. This was because molecular segmental motion occurred more easily at RT than at 77 K, thus dissipating more impact energy and exhibiting higher impact strength.

## 4. Conclusions

In this study, BSEA was utilized to efficiently improve the LOC and the comprehensive mechanical properties of EP at RT and cryogenic conditions. From the LOC test results, the EP/BCI/BSEA-4 and EP/BCI/BSEA-5 composites showed liquid oxygen compatibility. Moreover, the bending properties, fracture toughness and impact strength of BSEA-modified EP were greatly enhanced at RT and 77 K compared with pure EP. The bending strength (251.64 MPa) increased by 113.67%, the fracture toughness (2.97 MPa·m^1/2^) increased by 81.10%, and the impact strength (31.85 kJ·m^−2^) increased by 128.81% compared with pure EP at 77 K. All the above results indicate that BSEA-modified EP exhibits broad potential applications in the field of CFRP with high-performance equipment and liquid oxygen tanks.

## Figures and Tables

**Table 1:** Formulations of the pure EP and EP composites.

Samples	E-44 (wt%)	BCI (wt%)	BSEA (wt%)	P (wt%)	DDM (g)
Pure EP	100	0	0	0	25
EP/BCI-25	75	25	0	0	31.25
EP/BCI/BSEA-2	73	25	2	0.18	30.75
EP/BCI/BSEA-3	72	25	3	0.27	30.50
EP/BCI/BSEA-4	71	25	4	0.35	30.25
EP/BCI/BSEA-5	70	25	5	0.44	30.00

**Table 2:** Impact sensitivity phenomena of modified EP.

Samples	Experimental Phenomena (Number of Times)				Total Number of Tests	IRS (%)
	Burning	Explosion	Flash	Charring		
Pure EP	0	1	2	0	20	10.5
EP/BCI-25	0	1	2	0	20	10.5
EP/BCI/BSEA-2	1	0	0	1	20	7.0
EP/BCI/BSEA-3	0	0	0	2	20	4.0
EP/BCI/BSEA-4	0	0	0	0	20	0
EP/BCI/BSEA-5	0	0	0	0	20	0

**Table 3:** C1s peak fitting results of pure EP and BSEA-modified EP.

Sample			C1s			
			COOR	C=O	C-O-C/C-OH/ C-N	C-C/C=C/ C-H
Pure EP	Before	BE (eV)	—	287.99	285.90	284.15
		Area (%)	—	1.22	13.86	84.92
	After explosion	BE (eV)	289.99	288.20	285.43	283.93
		Area (%)	2.46	3.36	24.80	69.38
EP/BCI/BSEA-2	Before	BE (eV)	—	287.65	285.16	283.71
		Area (%)	—	2.84	21.37	75.79
	After burning	BE (eV)	290.40	287.70	285.45	284.02
		Area (%)	1.50	4.67	14.52	79.31
EP/BCI/BSEA-5	Before	BE (eV)	—	287.70	285.10	283.76
		Area (%)	—	2.44	25.23	72.33
	After no reaction	BE (eV)	—	287.35	285.42	283.98
		Area (%)	—	6.47	15.51	78.02

**Table 4:** O1s and P2p peak fitting results of pure EP and BSEA-modified EP.

Sample			O1s		P2p		
			C-O-C	C=O	PO_3_/P_2_O_5_	P=O	P-O-C
EP/BCI/BSEA-2	Before	BE (eV)	531.95	531.20	—	—	—
		Area (%)	41.56	58.44	—	—	—
	After burning	BE (eV)	532.25	531.50	134.60	133.00	132.00
		Area (%)	33.83	66.17	24.76	57.34	17.90
EP/BCI/BSEA-5	Before	BE (eV)	531.80	531.25	—	—	—
		Area (%)	49.03	50.97	—	—	—
	After no reaction	BE (eV)	532.10	531.45	134.50	133.00	132.01
		Area (%)	35.08	64.92	24.46	44.43	31.11

## Data Availability

The data presented in this study are available on request from the corresponding author.

## Supplementary Materials

The following supporting information can be downloaded at: https://www.mdpi.com/article/10.3390/polym14204343/s1, Figure S1: O1s spectra of pure EP (a) before and (a1) after the liquid oxygen compatibility test; N1s spectra of pure EP (b) before and (b1) after, EP/BCI/BSEA-2 (c) before and (c1) after, EP/BCI/BSEA-5 (d) before and (d1) after the liquid oxygen compatibility test; Table S1: The bending properties of EP samples at RT and 77K; Table S2: The fracture toughness and impact strength of EP samples at RT and 77K; Video S1: burning; Video S2: no reaction.

## References

[1] Liu N., Wang H., Ma B., Xu B., Qu L., Fang D., Yang Y. (2022). Enhancing cryogenic mechanical properties of epoxy resins toughened by biscitraconimide resin. Compos. Sci. Technol..

[2] Wang H., Li S., Yuan Y., Liu X., Sun T., Wu Z. (2019). Study of the epoxy/amine equivalent ratio on thermal properties, cryogenic mechanical properties, and liquid oxygen compatibility of the bisphenol A epoxy resin containing phosphorus. High Perform. Polym..

[3] Tapeinos I.G., Zarouchas D.S., Bergsma O.K., Koussios S., Benedictus R. (2019). Evaluation of the mechanical performance of a composite multi-cell tank for cryogenic storage: Part I—Tank pressure window based on progressive failure analysis. Int. J. Hydrogen Energy.

[4] Liu N., Ma B., Liu F., Huang W., Xu B., Qu L., Yang Y. (2021). Progress in research on composite cryogenic propellant tank for large aerospace vehicles. Compos. Part A Appl. Sci. Manuf..

[5] Morino Y., Shimoda T., Morimoto T., Ishikawa T., Aoki T. (2001). Applicability of CFRP materials to the cryogenic propellant tank for reusable launch vehicle (RLV). Adv. Compos. Mater..

[6] Wang H., Li C., Hou Z., Li B., Cai H. (2022). A phosphorus-containing imidazole derivative towards the liquid oxygen compatibility and toughness of epoxy resin. RSC Adv..

[7] Huang C., Lei Y.J. (2015). Research Progress on Design of Composite Cryogenic Tank in Large Launch Vehicle. Aerosp. Mater. Technol..

[8] Qi Y., Jiang D., Ju S., Zhang J., Cui X. (2019). Determining the interphase thickness and properties in carbon fiber reinforced fast and conventional curing epoxy matrix composites using peak force atomic force microscopy. Compos. Sci. Technol..

[9] Shi X.-H., Chen L., Zhao Q., Long J.-W., Li Y.-M., Wang Y.-Z. (2020). Epoxy resin composites reinforced and fire-retarded by surficially-treated carbon fibers via a tunable and facile process. Compos. Sci. Technol..

[10] Kim J., Cha J., Chung B., Ryu S., Hong S.H. (2020). Fabrication and mechanical properties of carbon fiber/epoxy nanocomposites containing high loadings of noncovalently functionalized graphene nanoplatelets. Compos. Sci. Technol..

[11] Zhan L., Guan C., Huang C., Yang X. (2019). Analysis of research status of composite cryotank for space. Aeronaut Manuf Technol.

[12] Peng C., Li J., Wu Z., Peng W., Zhou D. (2016). Investigating into the liquid oxygen compatibility of a modified epoxy resin containing silicon/phosphorus and its mechanical behavior at cryogenic temperature. RSC Adv..

[13] Rankin S.M., Moody M.K., Naskar A.K., Bowland C.C. (2020). Enhancing functionalities in carbon fiber composites by titanium dioxide nanoparticles. Compos. Sci. Technol..

[14] Karbhari V.M., Xian G., Hong S. (2021). Effect of thermal exposure on carbon fiber reinforced composites used in civil infrastructure rehabilitation. Compos. Part A Appl. Sci. Manuf..

[15] Liang J., Bai M., Gu Y., Wang S., Li M., Zhang Z. (2021). Enhanced electromagnetic shielding property and anisotropic shielding behavior of corrugated carbon fiber felt composite and its sandwich structure. Compos. Part A Appl. Sci. Manuf..

[16] Qu L., Zhang C., Li P., Dai X., Xu T., Sui Y., Gu J., Dou Y. (2018). Improved thermal properties of epoxy resin modified with polymethyl methacrylate-microencapsulated phosphorus-nitrogen-containing flame retardant. RSC Adv..

[17] Qu L., Sui Y., Zhang C., Li P., Dai X., Xu B., Fang D. (2019). POSS-functionalized graphene oxide hybrids with improved dispersive and smoke-suppressive properties for epoxy flame-retardant application. Eur. Polym. J..

[18] Liu X.-F., Liu B.-W., Luo X., Guo D.-M., Zhong H.-Y., Chen L., Wang Y.-Z. (2019). A novel phosphorus-containing semi-aromatic polyester toward flame retardancy and enhanced mechanical properties of epoxy resin. Chem. Eng. J..

[19] Auvergne R., Caillol S., David G., Boutevin B., Pascault J.-P. (2013). Biobased Thermosetting Epoxy: Present and Future. Chem. Rev..

[20] Liu L., Xu Y., Xu M., Li Z., Hu Y., Li B. (2019). Economical and facile synthesis of a highly efficient flame retardant for simultaneous improvement of fire retardancy, smoke suppression and moisture resistance of epoxy resins. Compos. Part B Eng..

[21] Jin F.-L., Li X., Park S.-J. (2015). Synthesis and application of epoxy resins: A review. J. Ind. Eng. Chem..

[22] Cai H., Hu J., Wang Y., Wang J. (2019). Liquid oxygen compatibility and toughness of epoxy resin modified by a novel hyperbranched polysiloxane. Mater. Res. Express.

[23] Biswas B., Kandola B.K. (2011). The effect of chemically reactive type flame retardant additives on flammability of PES toughened epoxy resin and carbon fiber-reinforced composites. Polym. Adv. Technol..

[24] Feng Q., Yang J., Liu Y., Xiao H., Fu S.-Y. (2014). Simultaneously Enhanced Cryogenic Tensile Strength, Ductility and Impact Resistance of Epoxy Resins by Polyethylene Glycol. J. Mater. Sci. Technol..

[25] Yi X.F., Mishra A.K., Kim N.H., Ku B.-C., Lee J.H. (2013). Synergistic effects of oxidized CNTs and reactive oligomer on the fracture toughness and mechanical properties of epoxy. Compos. Part A Appl. Sci. Manuf..

[26] Mill T., Chamberlain D., Stringham R., Kirshen N., Irwin K. (1970). Investigation of the Reactivity of Organic Materials in Liquid Oxygen.

[27] Li S., Li J., Cui Y., Ye J., Chen D., Yuan Y., Liu X., Liu M., Peng C., Wu Z. (2021). Liquid oxygen compatibility of epoxy matrix and carbon fiber reinforced epoxy composite. Compos. Part A Appl. Sci. Manuf..

[28] Bowden F.P., Yoffe A.D. (1952). The Initiation and Growth of Explosions in Liquids and Solids. Aeronaut. J..

[29] Amster A., Chamberlain D., Schulenberg F., Stringham R. (1967). Investigation of Reactivity of Launch Vehicle Materials with Liquid Oxygen Annual Report.

[30] Ai Y.-F., Xia L., Pang F.-Q., Xu Y.-L., Zhao H.-B., Jian R.-K. (2020). Mechanically strong and flame-retardant epoxy resins with anti-corrosion performance. Compos. Part B Eng..

[31] Xie W., Huang S., Tang D., Liu S., Zhao J. (2020). Correction: Synthesis of a furfural-based DOPO-containing co-curing agent for fire-safe epoxy resins. RSC Adv..

[32] Salmeia K.A., Gaan S. (2015). An overview of some recent advances in DOPO-derivatives: Chemistry and flame retardant applications. Polymer Degrad. Stab..

[33] Jian R., Wang P., Xia L., Zheng X. (2017). Effect of a novel P/N/S-containing reactive flame retardant on curing behavior, thermal and flame-retardant properties of epoxy resin. J. Anal. Appl. Pyrolysis.

[34] Wu Z., Li J., Wang Z. (2014). Liquid oxygen compatibility and thermal stability of bisphenol A and bisphenol F epoxy resins modified by DOPO. Polym. Adv. Technol..

[35] Li J., Liu X., Wu Z., Wang Z. (2016). The effect of 10-(2, 5-dihydroxyphenyl)-9, 10-dihydro-9-oxa-10-phosphaphenanthrene-10-oxide on liquid oxygen compatibility and cryogenic mechanical properties of epoxy resins. High Perform. Polym..

[36] Wang H., Peng C., Li S., Liu X., Wu Z. (2018). Improvement of the liquid oxygen compatibility of epoxy via the addition of surface-modified boehmite. J. Appl. Polym. Sci..

[37] Liu N., Wang H., Xu B., Qu L., Fang D. (2022). Cross-linkable phosphorus/nitrogen-containing aromatic ethylenediamine endowing epoxy resin with excellent flame retardancy and mechanical properties. Compos. Part A Appl. Sci. Manuf..

[38] Wu Z., Li S., Liu M., Wang H., Wang Z., Liu X. (2015). Study on liquid oxygen compatibility of bromine-containing epoxy resins for the application in liquid oxygen tank. Polym. Adv. Technol..

[39] Feng Y., He C., Wen Y., Ye Y., Zhou X., Xie X., Mai Y.-W. (2017). Improving thermal and flame retardant properties of epoxy resin by functionalized graphene containing phosphorous, nitrogen and silicon elements. Compos. Part A Appl. Sci. Manuf..

[40] Shi Y.-Q., Fu T., Xu Y.-J., Li D.-F., Wang X.-L., Wang Y.-Z. (2018). Novel phosphorus-containing halogen-free ionic liquid toward fire safety epoxy resin with well-balanced comprehensive performance. Chem. Eng. J..

[41] Li N., Zong L., Wu Z., Liu C., Wang X., Bao F., Wang J., Jian X. (2017). Amino-terminated nitrogen-rich layer to improve the interlaminar shear strength between carbon fiber and a thermoplastic matrix. Compos. Part A Appl. Sci. Manuf..

[42] Peng W., Nie S.-B., Xu Y.-X., Yang W. (2021). A tetra-DOPO derivative as highly efficient flame retardant for epoxy resins. Polym. Degrad. Stab..

[43] Zou J., Duan H., Chen Y., Ji S., Cao J., Ma H. (2020). A P/N/S-containing high-efficiency flame retardant endowing epoxy resin with excellent flame retardance, mechanical properties and heat resistance. Compos. Part B Eng..

[44] Duan H., Chen Y., Ji S., Hu R., Ma H. (2019). A novel phosphorus/nitrogen-containing polycarboxylic acid endowing epoxy resin with excellent flame retardance and mechanical properties. Chem. Eng. J..

