# Supplementary material for: Liquid Oxygen Compatibility and Ultra-Low-Temperature Mechanical Properties of Modified epoxy Resin Containing Phosphorus and Nitrogen
Source: Polymers (Basel). 2022 Oct 15;14(20):4343. doi: 10.3390/polym14204343 (PMC9610556; doi:10.3390/polym14204343)
Supplement: Supplementary file 1 [file polymers-14-04343-s001.zip › polymers-1961380-supplementary.pdf]

polymers

Liquid Oxygen Compatibility and Ultra-Low-Temperature Mechanical Properties of Modified Epoxy Resin Containing Phosphorus and Nitrogen

Ni Liu [1,2], Hui Wang [1,2], Shun Wang [1,2], Baosheng Xu [1,2,*] and Lijie Qu [1,2,*]

[1] Institute of Advanced Structure Technology, Beijing Institute of Technology, Beijing 100081, China
[2] Beijing Key Laboratory of Lightweight Multi-Functional Composite Materials and Structures, Beijing Institute of Technology, Beijing 100081, China
* Correspondence: xubsh@bit.edu.cn (B.X.); qulijie@bit.edu.cn (L.Q.)

Supplementary information:

Table S1. The bending properties of EP samples at RT and 77K.

Samples	Bending strength (MPa)		Bending modulus		Bending deflection (mm)	
	RT	77K	RT	77K	RT	77K
Pure EP	150.96±1.85	117.77±2.18	3.24±0.24	6.78±0.55	4.61±0.42	2.65±0.07
EP/BCI-25	209.08±4.42	202.36±3.31	2.65±0.05	7.13±0.09	10.68±0.20	3.56±0.12
EP/BCI/BSEA-2	163.10±3.26	251.64±11.36	3.23±0.04	6.82±0.11	11.66±0.71	5.50±0.46
EP/BCI/BSEA-3	159.00±2.93	246.16±8.27	2.95±0.11	6.65±0.51	12.36±0.92	5.25±0.52
EP/BCI/BSEA-4	143.90±1.69	219.09±7.78	2.93±0.08	6.63±0.20	14.84±1.12	4.79±0.06
EP/BCI/BSEA-5	131.86±2.13	202.10±9.19	3.12±0.07	6.52±0.19	17.67±1.31	4.49±0.04

Table S2. The fracture toughness and impact strength of EP samples at RT and 77K.

Samples	K_{IC} (MPa·m$^{1/2}$)		Impact strength (KJ·m^{-2})	
	RT	77K	RT	77K
Pure EP	0.92±0.07	1.64±0.03	24.00±1.50	13.92±0.58
EP/BCI-25	1.77±0.08	2.29±0.02	61.24±7.48	28.96±2.33
EP/BCI/BSEA-2	1.80±0.17	2.81±0.03	72.44±19.43	31.85±9.43
EP/BCI/BSEA-3	1.82±0.02	2.90±0.08	59.58±12.79	20.64±9.22
EP/BCI/BSEA-4	2.09±0.01	2.97±0.15	41.91±11.84	15.45±1.22
EP/BCI/BSEA-5	1.94±0.08	2.92±0.10	37.5±2.43	11.25±0.81

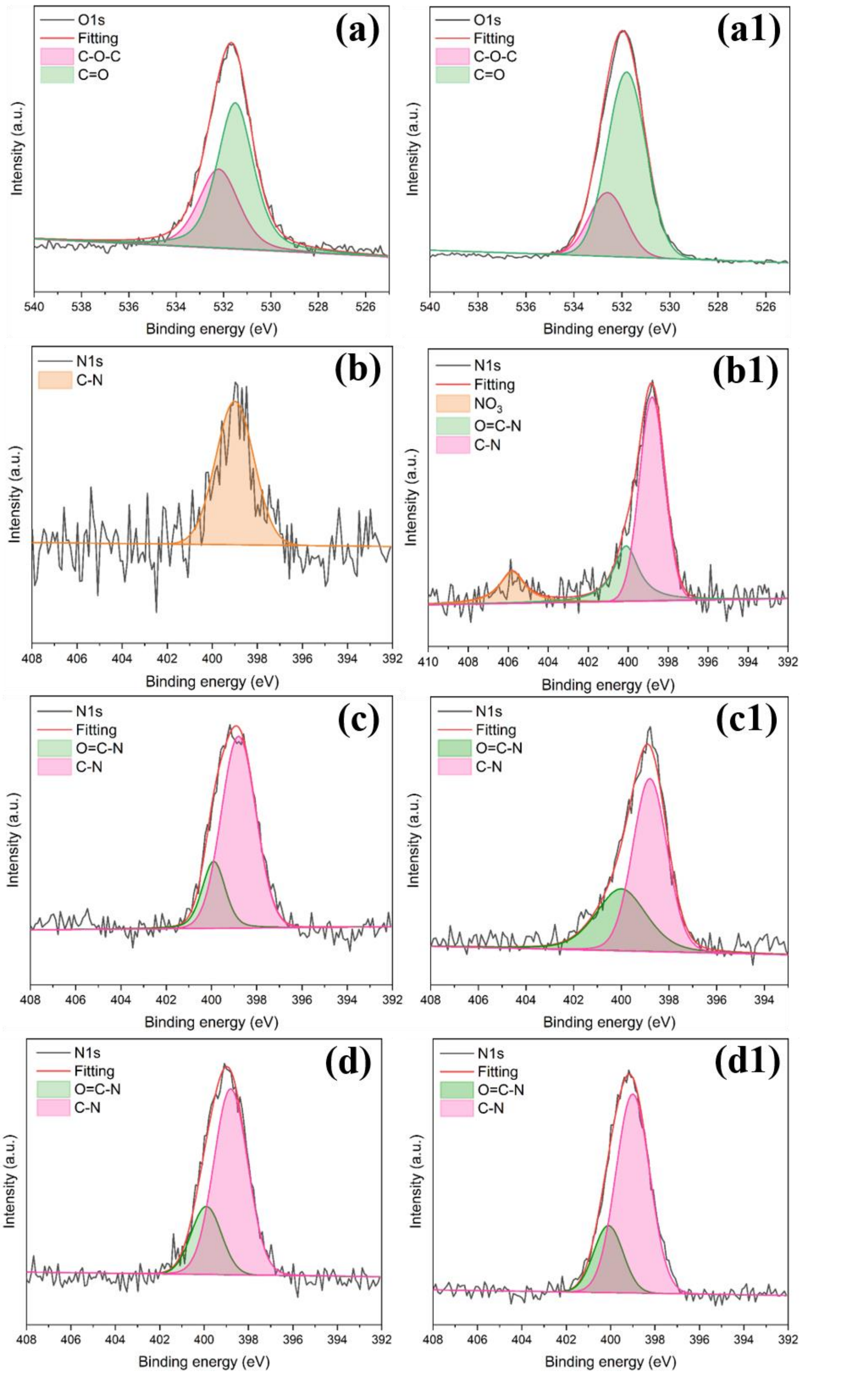

Figure S1. O1s spectra of pure EP (a) before and (a1) after the liquid oxygen compatibility test; N1s spectra of pure EP (b) before and (b1) after, EP/BCI/BSEA-2 (c) before and (c1) after, EP/BCI/BSEA-5 (d) before and (d1) after the liquid oxygen compatibility test.
